# Effect of Emodin on Preventing Postoperative Intra-Abdominal Adhesion Formation

**DOI:** 10.1155/2017/1740317

**Published:** 2017-08-02

**Authors:** Guangbing Wei, Yunhua Wu, Qi Gao, Cancan Zhou, Kai Wang, Cong Shen, Guanghui Wang, Kang Wang, Xuejun Sun, Xuqi Li

**Affiliations:** ^1^Department of General Surgery, The First Affiliated Hospital of Xi'an Jiaotong University, Xi'an, Shaanxi 710061, China; ^2^Department of Hepatobiliary Surgery, The First Affiliated Hospital of Xi'an Jiaotong University, Xi'an, Shaanxi 710061, China; ^3^Department of Medical Imaging, The First Affiliated Hospital of Xi'an Jiaotong University, Xi'an, Shaanxi 710061, China

## Abstract

**Background:**

Postoperative intra-abdominal adhesions are a major complication after abdominal surgery. Although various methods have been used to prevent and treat adhesions, the effects have not been satisfactory. Emodin, a naturally occurring anthraquinone derivative and an active ingredient in traditional Chinese herbs, exhibits a variety of pharmacological effects. In our study, we demonstrated the effect of emodin treatment on preventing postoperative adhesion formation.

**Materials and Methods:**

A total of 48 rats were divided into six groups. Abdominal adhesions were created by abrasion of the cecum and its opposite abdominal wall. In the experimental groups, the rats were administered daily oral doses of emodin. On the seventh day after operation, the rats were euthanized, and blood and pathological specimens were collected. Abdominal adhesion formation was evaluated by necropsy, pathology, immunohistochemistry, Western blot, and enzyme-linked immunosorbent assay analyses.

**Results:**

Abdominal adhesions were markedly reduced by emodin treatment. Compared with the control group, collagen deposition was reduced and the peritoneal mesothelial completeness rate was higher in the emodin-treated groups. Emodin had anti-inflammatory effects, reduced oxidative stress, and promoted the movement of the intestinal tract (*P* < 0.05).

**Conclusion:**

Emodin significantly reduced intra-abdominal adhesion formation in a rat model.

## 1. Introduction

Intra-abdominal adhesion formation is a major complication after abdominal surgery. Patients have a 90%–95% risk of developing intraperitoneal adhesions after laparotomy [1, 2], which is one of the most upsetting complications after gastrointestinal surgery. Peritoneal adhesions can cause various problems, such as small-bowel obstruction, female infertility, chronic abdominal pain, and increased difficulty during reoperation, all of which greatly influence quality of life and increase medical costs [3–5]. The strategies to treat and prevent adhesions can be divided into four categories: general principles, surgical techniques, chemical agents, and mechanical barriers [6, 7]. Although several methods have been applied to prevent adhesion formation, a “gold standard” for treatment has not been determined yet, and surgeons still need to seek more effective methods to prevent and treat postoperative abdominal adhesions [8].

The formation of abdominal adhesions is a complex process that involves inflammation, angiogenesis, fibrinolysis, peritoneal tissue repair, and other biochemical events [4, 9–11]. When injury or trauma occurs in the abdominal cavity, ischemia of the local area occurs and inflammatory and coagulation cascades are activated within a few minutes. Inflammatory cells, such as neutrophils and macrophages, and tissue repair cells will migrate to the injured area, and the coagulated blood will form a fibrin mesh [12, 13]. Then, after approximately 24 hours, the mesothelium will start to grow, followed by fibroblast proliferation on day 3 and angiogenesis on day 5. If the peritoneum repairs well and the fibrin mesh is absorbed, the adhesions are very minor; otherwise, abdominal adhesions will form [2, 4]. Fibroblasts and the inflammatory system play major roles in the mechanism of abdominal adhesion formation [2, 14, 15]. Intestinal movements also play an important role in the formation of abdominal adhesion [16]. Early movement of the bowel disrupts the fibrin bridges and inhibits fibroblast invasion into the adhesive tissues. Additionally, mobilization encourages fibrinolysis through increased fluid movement and metabolite exchange in the peritoneum [16, 17].

Emodin (1,3,8-trihydroxy-6-methylanthraquinone) is a naturally occurring anthraquinone derivative and an active ingredient in traditional Chinese herbs, including *Rheum palmatum*, *Polygonum cuspidatum*, *Polygonum multiflorum*, *Aloe vera*, and *Cassia obtusifolia*. Emodin exhibits a variety of pharmacological benefits in pharmacological studies [18]. According to previous studies [18–21], emodin has been demonstrated to have various effects, including antiviral, antibacterial, antiallergenic, antiosteoporotic, antidiabetic, anti-inflammatory, and antitumor effects, and can reduce oxidative stress. Moreover, emodin can decrease collagen deposition in pancreatitis and pulmonary injuries and downregulate the TGF-*β* signaling pathway in many human cancers [20, 22]. As a laxative drug in traditional Chinese herbs [23], the primary effect of emodin is promoting intestinal movement. Since inflammation, collagen deposition, oxidative stress, and intestinal movement all play important roles in postoperative intra-abdominal adhesion formation, we speculated that emodin may reduce postoperative adhesion formation. In this study, we intended to demonstrate that emodin can prevent postoperative abdominal adhesion formation.

## 2. Materials and Methods

### 2.1. Animals and Chemicals

A total of 48 Sprague-Dawley rats weighing 200 to 250 g were purchased from the Experimental Animal Center of Xi'an Jiaotong University. The animals were treated in a humane manner in accordance with the Declaration of Helsinki. All animals were fed ad libitum with a commercial diet and had continuous access to fresh water. The animals were housed under standard laboratory conditions at 22 ± 2°C. This experiment was accomplished in the Xi'an Jiaotong University Experimental Research Laboratory with the consent of the Experimental Animals Ethics Committee [24].

Emodin (PubChem CID: 10207) was purchased from Sigma-Aldrich Co. LLC® (St. Louis, MO) and was dissolved in 0.5% sodium carboxymethyl cellulose (0.5% CMC-Na®; Henan Qianzhi Company, Henan, China) at different concentrations [25].

### 2.2. Study Design and Surgical Procedure

All rats were fasted, and the hair on their abdomen was removed one day before surgery. The rats were equally divided into six groups. The animals were deeply anesthetized by an intraperitoneal injection of 50 mg/kg barbital sodium (Guidechem, Shanghai, China), and the abdominal skin was disinfected with povidone-iodine before the operation. As previously described [3, 26, 27], a vertical midline incision (2-3 cm long) was made, except in the animals with previous intra-abdominal adhesion formation. Excluding the sham operation group, the anterior surface of each cecum was scraped with a soft swab 40 times, which induced slight serosal hemorrhage resulting in the formation of surface lesions in an area of approximately 1.5 cm × 1.5 cm. The rat abdominal wall opposite to the scratched cecum was treated in the same manner. Prior to closing the abdominal cavity, the cecum was placed in its original position opposite to the wounded abdominal wall in full contact with each other. In the sham operation group, the rats did not undergo the abdominal adhesion formation procedure. In the sodium hyaluronate group, 2 mL of medical-grade hyaluronate gel (Qingdao Haitao Biochemical Co. Ltd., Qingdao, China) was daubed on the abraded area before closing the abdominal cavity. The abdomen was closed in two layers using interrupted 3-0 Vicryl® sutures. After the operation, the rats in the three experimental groups were orally administered 20 (low-dose group), 40 (middle-dose group), or 80 (high-dose group) mg/kg emodin daily. The sham operation group and the control group were orally administered the same amount of 0.5% CMC-Na once per day for one week.

### 2.3. Adhesion Grading and Assessment

One week after surgery, all animals were anesthetized as previously described, and a reverse U incision was performed to assess the adhesions. Adhesion formation was measured by two independent researchers who were blinded to the study protocol, according to the method described by Hoffmann et al. [28] and Lauder et al. [29]. The Hoffmann's scoring scheme considers the number, strength, and distribution of adhesions. Lauder's schemes were measured and expressed as a percent of the total deperitonealized surface area [7] (Supplementary Tables 1 and 2 available online at https://doi.org/10.1155/2017/1740317). After adhesion grading was performed, the tissues and blood serum were collected for the next experiments.

### 2.4. Histopathological Evaluation

Hematoxylin and eosin staining was used to evaluate the inflammation and fibrillation condition. After 24 hours of fixation, the adhesion tissues of the injured cecum wall and parietal peritoneum were embedded in paraffin and then cut into 4 *μ*m thick serial paraffin sections. A portion of the paraffin sections was stained by hematoxylin and eosin to observe the morphology and adhesion conditions of the tissues. Histopathological evaluations of fibrosis and inflammation were made by light microscopy. The stained sections were evaluated by two pathologists from the Pathology Department of the First Affiliated Hospital of Xi'an Jiaotong University who were blinded to the experimental groups. At least five randomly selected high-power fields were reviewed for each section, and at least four sections per rat were evaluated and graded using the scoring system described in Supplementary Table 3 [30, 31].

### 2.5. Masson Staining for Collagen Deposition

A subset of the paraffin sections was used for Masson staining to observe the collagen content. The paraffin sections were stained using a Masson staining kit (Bogoo Biotechnology Co. Ltd., Shanghai, China) following the manufacturer's instructions. The percentage of positive staining was assessed by the Image-Pro Plus 5.0 software (Leica Qwin Plus, Leica Microsystem Imaging Solutions Ltd., Cambridge, UK). Eight microscopic fields were randomly selected to measure the average collagen thickness in the adhesive tissues.

### 2.6. Immunohistochemical Staining

Immunohistochemical staining was used to assess collagen deposition (*α*-SMA, MMP-9), mesothelial cell healing (CK-18), and gastrointestinal dynamics (C-kit) and was performed using a streptavidin-biotin kit (Maxim, Fuzhou, China) following the manufacturer's instructions. The sections were deparaffinized and rehydrated, incubated with 30 g/L hydrogen peroxide solution at room temperature for 5 minutes, and blocked with goat serum. Subsequently, the sections were incubated with mouse anti-rat *α*-smooth muscle actin (*α*-SMA; 1 : 100 dilution, Santa Cruz Biotechnology, Dallas, TX, USA), tyrosine kinase receptor (C-kit; 1 : 100 dilution, Abcam, UK), cytokeratin-18 (CK-18; 1 : 50 dilution, Abcam, UK), and matrix metalloproteinase-9 antibodies (MMP-9; 1 : 100 dilution, Abcam) at 4°C overnight. The sections were then incubated with biotinylated rabbit anti-mouse IgG for 20 minutes. Incubation with streptavidin-biotin peroxidase complex at 37°C was performed for another 20 minutes. The sections were washed in phosphate-buffered saline four times for 5 minutes per wash. Diaminobenzidine tetrahydrochloride was used for visualization, and hematoxylin was used as the counterstain. The sections were dehydrated, mounted, and sealed. To evaluate the expression of these indicators, at least five random high-power fields of adhesion tissue were reviewed for each section as described in the histopathological evaluation. Then, the CK-18 stain was used to assess the completeness rate of the mesothelial cells. The rate was calculated by the stained mesothelial cell length in the adhesion tissue divided by the total mesothelial cell length in the selected fields. The *α*-SMA, C-kit, and MMP-9 scoring system is as follows: 0—no expression, 1—low expression, 2—moderate expression, 3—strong expression, and 4—very strong expression. The expression condition in the adhesive tissue was calculated as the average score of examined sections.

### 2.7. Western Blot

Western blot was performed according to previous studies [24, 32] to assess inflammation (COX-2) and collagen deposition (*α*-SMA, MMP-9) to further delineate the mechanism of emodin (SMAD3). Mammalian protein lysis buffer (Thermo Fisher Scientific, Waltham, MA, USA) was used to extract the total tissue protein. The same amounts of protein were subjected to sodium dodecyl sulfate-polyacrylamide gel electrophoresis and then transferred to polyvinylidene fluoride membranes (EMD Millipore, Billerica, MA, USA). The membranes were incubated in the diluted primary antibodies overnight and stored at 4°C. The primary antibodies were anti-COX-2 antibody (Santa Cruz, 1 : 200 dilution), anti-SMAD3 antibody (Santa Cruz, 1 : 500 dilution), anti-*α*-SMA antibody (Santa Cruz, 1 : 200 dilution), and anti-beta-actin (*β*-actin) antibody (Santa Cruz, 1 : 1000 dilution). The membranes were incubated with the secondary antibody followed by horseradish peroxidase (HRP; Santa Cruz). Then, an enhanced chemiluminescence system (EMD Millipore) was used to detect the bands. The intensity of the bands was calculated using Image-Pro Plus 5.0 software (Media Cybernetics Inc., Rockville, MD, USA).

### 2.8. Enzyme-Linked Immunosorbent Assay (ELISA)

Interleukin-6 (IL-6) and transforming growth factor-*β*1 (TGF-*β*1) (indicators of inflammation) as well as gastrin and motilin (indicators of gastrointestinal dynamics) levels were measured in blood that was collected 7 days after surgery. Four commercial ELISA kits (all from Meilian Biology, Shanghai, China) for IL-6, TGF-*β*1, gastrin, and motilin were used according to the manufacturers' protocols. The concentrations of the samples were calculated using a standard curve. The levels of the assessed indicators are expressed as picograms per milligram of protein.

### 2.9. Chemiluminescence Test for Reactive Oxygen Species

As reported previously [33], reactive oxygen species (ROS) were measured in the peritoneal lavage fluid 7 days after the operation. The lavage fluids were collected before opening the abdominal cavity. A commercial ROS detection kit (Sinovac Biochemical Reagents, Shanghai, China) was used according to the manufacturer's protocol. The concentrations of the samples were calculated using a standard curve.

### 2.10. Gastrointestinal Dynamics Experiment

An ink-propelling test was performed to determine the condition of the gastrointestinal dynamics in the different groups. All rats were administered 2 mL of ink, consisting of 5% carbon powder and a 0.5% CMC-Na suspension, by oral gavage 30 minutes before the second laparotomy. After assessing the adhesion condition, the total small bowel was removed from the abdominal cavity. Then, the small intestine was cut along the antimesenteric side. Next, we measured the total length from the distal stomach to the ileocecal junction and the length from the pylorus to the furthest point that the carbon powder had reached. The intestinal propulsion rate was calculated using the following equation: intestinal propulsion rate (%) = length of carbon powder movement/total length of small intestine × 100%.

### 2.11. Statistical Analyses

All data were analyzed with the SPSS18.0 software (Chicago, IL, USA) and are presented as means ± standard errors of the mean (SEM). Analysis of variance (ANOVA) was performed to determine significant differences in normally distributed data, and the abnormally distributed data were analyzed by the Kruskal-Wallis test. Enumeration data were determined by Fisher's exact test. *P* values <0.05 were considered significant.

## 3. Results

### 3.1. Gross Observations Indicate That Emodin Treatment Reduced Abdominal Adhesions

No animals died during the operation or postoperative treatment; therefore, a total of 48 rats completed the study. No wound infections or disruptions occurred before the second laparotomy.

**Figure 1:**
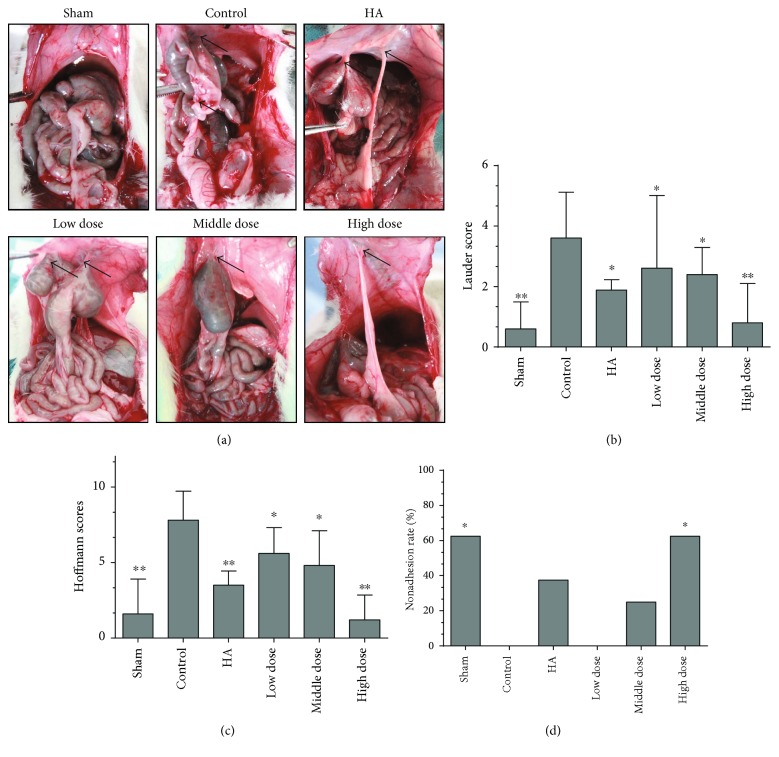
Emodin treatment prevents postoperative intra-abdominal adhesion formation in rats on the seventh day after the operation (*n* = 8). (a) The sham operation group (sham) rarely had intra-abdominal adhesions. Control group (control) animals developed a large number of extensive and thick adhesions that were difficult to separate. The sodium hyaluronate group (HA) had a few adhesion formations. The low-dose emodin group, middle-dose emodin group, and high-dose emodin group developed fewer adhesions than the control group. Black arrows indicate adhesions. (b) The Lauder scores of the different groups (total *P* = 0.0022 compared with the control group, ^∗^*P* < 0.05 and ^∗∗^*P* < 0.01, abnormally distributed, Kruskal-Wallis test). (c) The total Hoffmann scores of the different groups (total *P* < 0.0001 compared with the control group, ^∗^*P* < 0.05 and ^∗∗^*P* < 0.01, abnormally distributed, Kruskal-Wallis test). (d) The nonadhesion rates in the different groups (total *P* = 0.004 compared with the control group, ^∗^*P* < 0.05, Fisher's exact test).

To investigate the effect of emodin on preventing adhesion formation after surgery, we first evaluated adhesion formation using the scoring system as described before when the abdominal cavity was opened 7 days after the operation (Figure 1(a)). There were significant differences between the groups, and the adhesions were remarkably relieved by emodin treatment, especially in the high-dose emodin group (*P* < 0.05) (Figures 1(b) and 1(c); *P* = 0.0022 and *P* < 0.0001, resp.). Additionally, the highest nonadhesion rates were found in the sham operation and high-dose emodin groups (*P* = 0.011) (Figure 1(d)). Therefore, our gross observations indicate that emodin effectively reduces intra-abdominal adhesion formation.

### 3.2. Emodin Inhibits Intra-Abdominal Collagen Deposition

**Figure 2:**
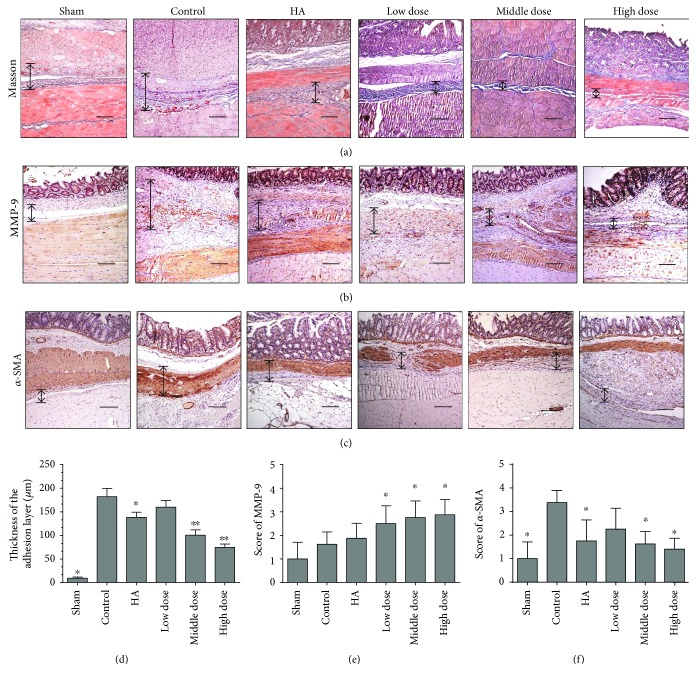
Emodin treatment reduced postoperative intra-abdominal collagen deposition seven days after operation. The magnification is 100× (*n* = 8; compared with the control group, ^∗^*P* < 0.05 and ^∗∗^*P* < 0.01). (a) Masson staining of the different groups (double-headed black arrows indicate adhesive tissues). (b) Immunohistochemical staining of MMP-9 among the different groups (double-headed black arrows indicate adhesive tissues). (c) Immunohistochemical staining of *α*-SMA (double-headed black arrows indicate adhesive tissue). (d) The score obtained from Masson staining shows that the thickness of the adhesion layer is thinner in the emodin-treated groups than in the other groups (*P* < 0.0001, abnormally distributed, Kruskal-Wallis test). (e) The score of MMP-9 staining among different groups (total *P* = 0.0005, abnormally distributed, Kruskal-Wallis test). (f) The score of *α*-SMA staining among different groups (total *P* = 0.0005, abnormally distributed, Kruskal-Wallis test).

Masson staining was used to assess collagen deposition in the adhesion tissues of different groups. The collagen layer was thinner in the emodin-treated groups (*P* < 0.0001) (Figures 2(a) and 2(d) and Supplementary Figure 1A).

To verify our results and to discover the possible mechanisms underlying the decrease in adhesive tissue collagen deposition after emodin treatment, we determined the MMP-9 and *α*-SMA expression levels using immunohistochemistry. Compared with the control group, MMP-9 levels were increased (*P* = 0.0003) (Figures 2(b) and 2(e) and Supplementary Figure 1B) and *α*-SMA levels were decreased (*P* = 0.0002) (Figures 2(c) and 2(f) and Supplementary Figure 1C) in the emodin-treated groups. These results suggest that emodin treatment inhibits collagen deposition.

### 3.3. Emodin Treatment Promotes Mesothelial Cell Healing

**Figure 3:**
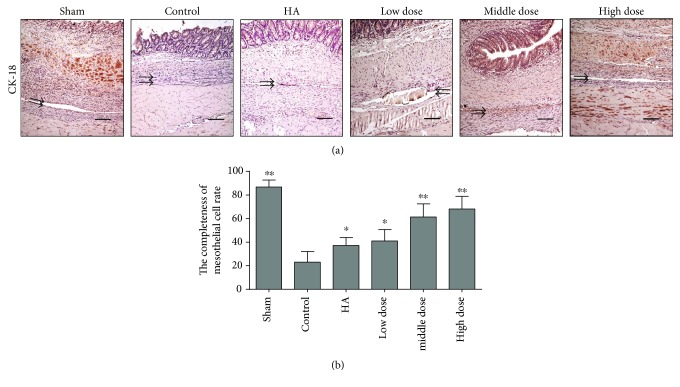
The completeness rate of normal mesothelial cells seven days after the operation (*n* = 8; compared with the control group, ^∗^*P* < 0.05 and ^∗∗^*P* < 0.01). (a) Immunohistochemical staining of CK-18 among the different groups. The magnification is 100× (black arrows indicate CK-18-positive mesothelial cells or injured mesothelial cell lines). (b) The emodin-treated groups had intact peritoneal mesothelial cell layers as detected by immunohistochemical staining for CK-18 (total *P* < 0.0001, abnormally distributed, Kruskal-Wallis test).

To evaluate healing of the peritoneal mesothelium, which plays an important role in abdominal adhesion formation, we used immunohistochemistry to determine the expression of cytokeratin in the peritoneal mesothelial cells of the different groups. In the groups treated with emodin, the peritoneal cells repaired the injured area more completely than those in the other groups (*P* < 0.0001) (Figures 3(a) and 3(b) and Supplementary Figure 1D). Emodin treatment promotes the process of mesothelial cell healing.

### 3.4. Emodin Treatment Alleviates Oxidative Stress

**Figure 4:**
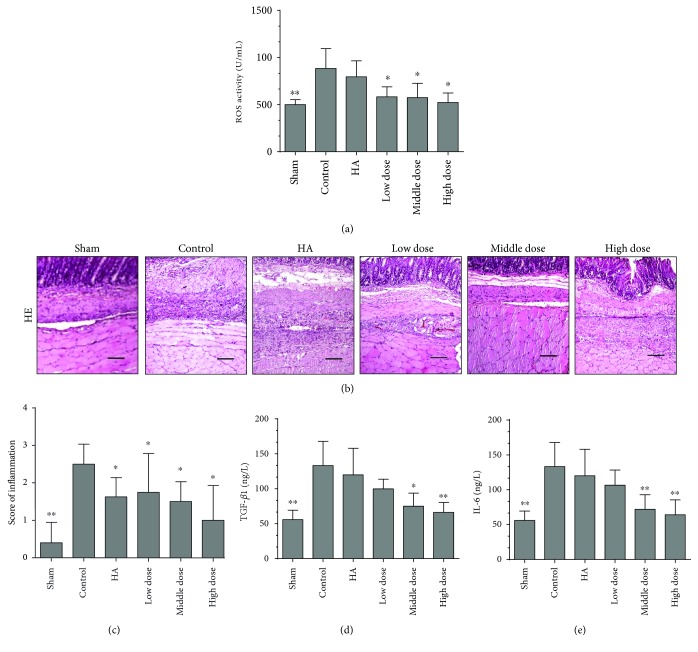
Emodin treatment alleviates oxidative stress and inhibits inflammation in rats seven days after the operation (*n* = 8; compared with the control group, ^∗^*P* < 0.05 and ^∗∗^*P* < 0.01). (a) Emodin-treated groups had lower expression levels of ROS (total *P* < 0.0001, abnormally distributed, Kruskal-Wallis test). (b) HE staining of the different groups. The magnification is 100×. (c) The scores of the inflammatory response determined by HE staining in the different groups (total *P* = 0.00177, abnormally distributed, Kruskal-Wallis test). (d) ELISA results showing the TGF-*β*1 levels in the blood on the seventh day after operation (total *P* < 0.0001, abnormally distributed, Kruskal-Wallis test). (e) ELISA results showing the IL-6 levels in the blood on the seventh day after operation (total *P* < 0.0001, abnormally distributed, Kruskal-Wallis test).

To determine additional mechanisms by which emodin treatment prevents abdominal adhesions, we determined the levels of ROS, indicators of oxidative stress reactions, in the peritoneal lavage fluids. The levels of ROS in the emodin-treated groups were lower than those in the control group (*P* < 0.0001) (Figure 4(a)). Therefore, a potential mechanism by which emodin prevents postoperative abdominal adhesions may be through alleviating oxidative stress reactions.

### 3.5. Emodin Treatment Inhibits Inflammation

**Figure 5:**
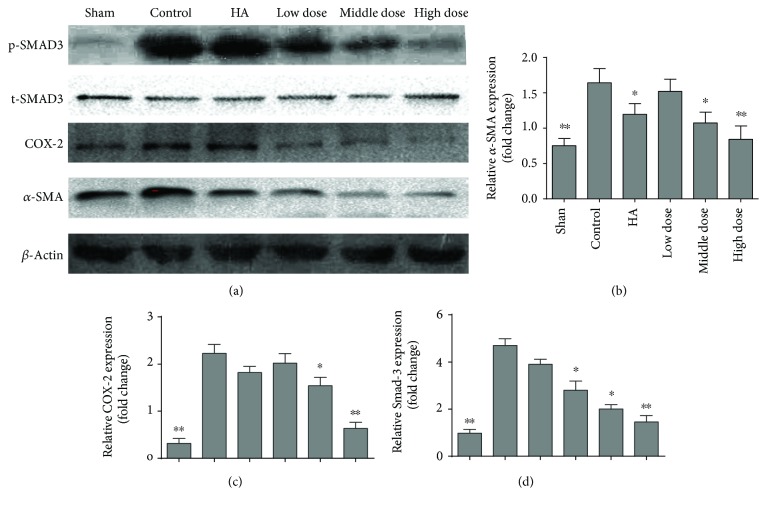
Western blot of SMAD-3, COX-2, and *α*-SMA in the different groups (*n* = 8; compared with the control group, ^∗^*P* < 0.05 and ^∗∗^*P* < 0.01, abnormally distributed, Kruskal-Wallis test). (a) Western blot of SMAD-3, COX-2, and *α*-SMA in the different groups. (b) The relative *α*-SMA expression of different groups (total *P* < 0.0001, abnormally distributed, Kruskal-Wallis test). (c) The relative COX-2 expression of different groups (total *P* < 0.0001, abnormally distributed, Kruskal-Wallis test). (d) The relative SMAD-3 expression of different groups (total *P* < 0.0001, abnormally distributed, Kruskal-Wallis test).

To confirm our observations and determine a possible mechanism by which emodin prevents postoperative intra-abdominal adhesion formation, we performed a macroscopic evaluation of the inflammatory response. Fibrosis was remarkably reduced and the inflammation score was lower in the emodin-treated groups than in the control group (*P* = 0.00177) (Figures 4(b) and 4(c) and Supplementary Figure 1E). We assessed the levels of TGF-*β* and IL-6 in the blood on the seventh day after the operation and found that there was less of an inflammatory reaction in the emodin-treated groups than in the controls (*P* < 0.0001) (Figures 4(d) and 4(e)). In addition, COX2, an important indicator of inflammation, was decreased in the emodin-treated groups, as shown in Figures 5(a) and 5(c) (*P* < 0.0001). To determine the probable mechanisms by which emodin reduces inflammation, we assessed the expression of SMAD-3, an activator of the TGF-*β* signaling pathway, in the different groups. Emodin treatment successfully decreased the expression of SMAD-3 (Figures 5(a) and 5(d), *P* < 0.0001). Thus, these results suggest that emodin potentially inhibits inflammation through a mechanism associated with the TGF-*β* signaling pathway.

### 3.6. Emodin Treatment Promotes Gastrointestinal Dynamics

**Figure 6:**
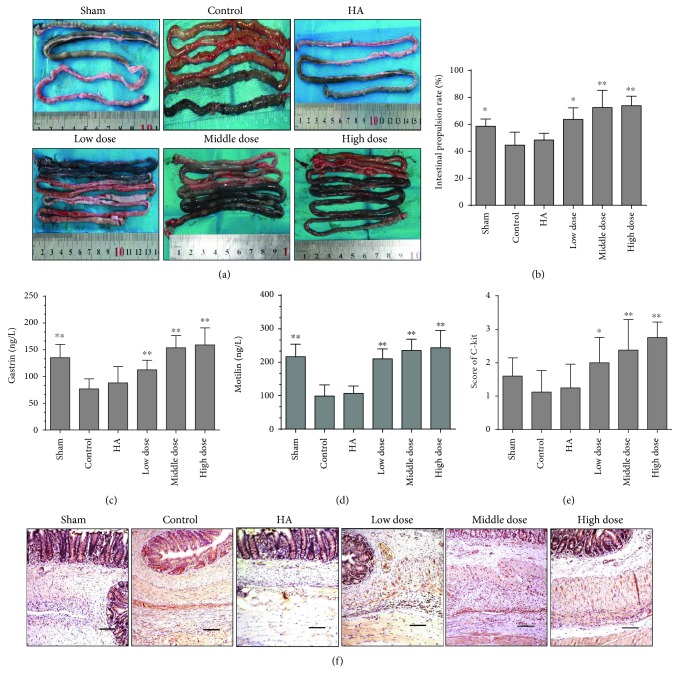
Gastrointestinal dynamics in the different groups (compared with the control group, ^∗^*P* < 0.05 and ^∗∗^*P* < 0.01). (a) Representative image of the ink-propelling test indicating intestinal movement conditions in different groups. (b) The intestinal propulsion rates determined by ink-propelling tests in the different groups (total *P* < 0.0001, abnormally distributed, Kruskal-Wallis test). (c) ELISA results showing gastrin levels in the blood on the seventh day after operation (total *P* < 0.0001, abnormally distributed, Kruskal-Wallis test). (d) ELISA results showing motilin levels in the blood on the seventh day after operation (total *P* < 0.0001, abnormally distributed, Kruskal-Wallis test). (e) The scores of C-kit expression in different groups (total *P* < 0.0001, abnormally distributed, Kruskal-Wallis test). (f) The immunohistochemical staining of C-kit expression in the different groups. The magnification is 100×.

To further determine the condition of the gastrointestinal dynamics in the different groups, we performed an ink-propelling test. The ink moved further in the emodin-treated groups, which suggests that emodin treatment promotes bowel movements (*P* < 0.0001) (Figures 6(a) and 6(b)). In addition to these gastrointestinal dynamic evaluations, we assessed several objective indicators to confirm our findings. By testing the levels of gastrin and motilin in the blood seven days after the operation, the emodin-treated groups had higher levels of gastrointestinal hormones than the other groups (*P* < 0.0001) (Figures 6(c) and 6(d)). Based on the immunohistochemical staining of C-kit, a specific maker for intestinal movement [34, 35], we drew the same conclusion (*P* < 0.0001) (Figures 6(e) and 6(f) and Supplementary Figure 1F). Thus, emodin treatment can promote gastrointestinal movement.

## 4. Discussion

Here, we demonstrated that emodin treatment could effectively reduce postoperative adhesion formation, reduce collagen formation, and accelerate healing of the peritoneal mesothelium. Emodin prevents adhesion formation in several ways, including the inhibition of inflammation, alleviation of oxidative stress, and promotion of intestinal tract movements. The results presented in this study strongly suggest that emodin can be an effective drug for the prevention of postoperative adhesion formation.

Emodin is a natural secondary plant product, originally isolated from the rhizomes of *Rheum palmatum*. In traditional Chinese medicine, emodin is used as an anti-inflammatory drug and to improve visceral stasis and promote movement of the gut [18, 36]. Emodin is a pleiotropic molecule capable of interacting with several major molecular targets, for example, TGF-*β*, NF-*κ*B, AKT/mTOR, and STAT3 [18]. We chose emodin as an antiabdominal adhesion drug because of the following reasons. In a previous study, emodin inhibited inflammation through TGF-*β* [37]. Emodin also reduced liver and pulmonary fibrosis [22, 38]. In addition to its properties as a laxative drug, emodin can promote intestinal movement [23]. While the formation of intestinal adhesions involves inflammation, collagen deposition, and oxidative stress, the TGF-*β* pathway also plays important roles in abdominal formation [11, 39]. Thus, we speculated that emodin may inhibit abdominal adhesion formation. In our study, the main effect of emodin was to prevent abdominal adhesion formation via blockage of the TGF-beta signaling pathway.

The fibrinolytic system plays a central role in the formation of postoperative intra-abdominal adhesions [1]. If complete fibrinolysis does not occur within 5 to 7 days after peritoneal injury, the temporary fibrin matrix will persist and gradually will become more organized as fibroblasts secrete collagen, which can lead to the formation of postoperative adhesions. In our study, we used Masson staining to detect collagen and immunohistochemistry and Western blot analyses to detect *α*-SMA. Emodin treatment reduced the collagen content in adhesion tissues. MMP-9, which can degrade the extracellular matrix and reduce collagen levels, was increased in the emodin-treated groups compared with the controls.

The process of peritoneal healing plays an important role in postoperative abdominal adhesion formation. If the peritoneal cells can repair the locally injured peritoneal area in time, then, adhesion formation will be reduced [6]. Cytokeratin-18 is a specific marker for mesothelial cells [40]. We compared the positive cytokeratin expression rates of mesothelial cells among the different groups, and the emodin-treated groups had a higher rate of expression in normal mesothelial cells. We concluded that the peritoneal mesothelial cells healed faster in the emodin-treated groups.

Adhesion formation stems from trauma/injury that results in the initiation of an inflammatory response, which leads to the formation of a fibrin matrix [3, 41]. There are various molecular targets that emodin may modulate. Emodin may possess a great potential as a therapeutic agent for a variety of inflammatory conditions. Emodin exerts anti-inflammatory effects on CIA mice through inhibition of the NF-*κ*B pathway [20, 42, 43]. In our study, through the evaluation of inflammatory scores in HE-stained sections and determination of TGF-*β*1 and IL-6 levels in blood and COX-2 expression in tissues, we concluded that emodin treatment decreased the inflammatory response. The potential mechanism of emodin in the reduction of inflammation may be through inhibition of the most important inflammatory pathway, the TGF-*β* signaling pathway. To investigate this possibility, we investigated the downstream molecule SMAD-3 [44]. Emodin treatment effectively decreased SMAD-3 expression. However, further study is required to fully elucidate the effects of emodin treatment on SMAD-3 expression.

Oxidative stress participates in the formation of postoperative abdominal adhesion formation, and inhibition of the oxidative stress pathway can be beneficial for the prevention of adhesion formation [45]. ROS are an indicator of oxidative stress. In our study, the expression of ROS was decreased in the emodin-treated groups, suggesting that one possible mechanism of emodin in preventing postoperative adhesion formation is by reducing oxidative stress.

The other possible mechanism for the reduction of postoperative adhesion formation by emodin may be via the promotion of gastrointestinal movement. As a laxative, emodin is traditionally used to treat constipation and can increase the contractility of intestinal smooth muscle [46]. Researchers have shown that stasis is a key element in the development of postoperative adhesions, and intestinal mobilization can acutely lyse adhesions and prevent adhesions from forming [16, 17]. In our study, we tested the gastrointestinal dynamics of the different groups by objective indicators and animal experiments. Emodin treatment markedly promoted the movement of the bowels. Intestinal movement could potentially reduce the probability of abdominal wall-to-bowel or bowel-to-bowel adhesion formation.

Importantly, increased intestinal motility may be associated with dehiscence following colonic anastomosis [47]. However, previous studies also proved that a moderate increase in intestinal movement does not increase the risk of anastomotic leakage [48]. In addition to this, according to the Enhanced Recovery After Surgery (ERAS®) guidelines for colonic surgery (colon resection and anastomosis) [49], patients are encouraged to take normal food as soon as possible after surgery and to drink immediately after recovery from anesthesia. Early food and fluid intake will inevitably promote intestinal motility [50]. Researchers have not observed an increase in anastomotic leakage among patients undergoing ERAS [51]. According to our study, increasing intestinal mobility has no significance on the development of anastomotic fistulas (Supplementary Figure 1C).

There were several limitations in this study. First, the mechanism of emodin with respect to adhesion reduction is still unclear. Second, we could not fully simulate the effects of emodin treatment on humans because we performed these experiments in animals. Thus, we could not ensure that the rat model accurately models the response in humans. Third, although emodin may be a new method for the treatment of postoperative abdominal adhesion formation, the side effects of this compound are unknown. In particular, toxicology and pharmacokinetic studies are warranted.

## 5. Conclusions

Emodin treatment exerts anti-inflammatory effects, alleviates oxidative stress, and promotes the movement of the intestinal tract. Therefore, emodin is a promising drug for the prevention of postoperative adhesion formation. However, the mechanisms of emodin treatment in preventing abdominal adhesions require further study.

## Supplementary Material

Supplementary Table 1. Adhesion scoring scheme of Lauder et al. Supplementary Table 2. Adhesion scoring scheme of Hoffmann et al. Supplementary Table 3. Histopathological criteria for adhesion scoring. Supplementary Figure 1. Immunohistochemical staining of Masson, MMP-9, α-SMA, CK-18, HE and C-kit of the different groups. The magnification is 200× (double-headed black arrows indicate adhesive tissues; black arrows indicate CK-18 positive mesothelial cells or injured mesothelial cell lines).
