# Supplementary material for: Effect of Emodin on Preventing Postoperative Intra-Abdominal Adhesion Formation
Source: Oxid Med Cell Longev. 2017 Aug 2;2017:1740317. doi: 10.1155/2017/1740317 (PMC5558648; doi:10.1155/2017/1740317)
Supplement: Supplementary file 1 — Supplementary Table 1. Adhesion scoring scheme of Lauder et al. Supplementary Table 2. Adhesion scoring scheme of Hoffmann et al. Supplementary Table 3. Histopathological criteria for adhesion scoring. Supplementary Figure 1. Immunohistochemical staining of Masson, MMP-9, α-SMA, CK-18, HE and C-kit of the different groups. The magnification is 200× (double-headed black arrows indicate adhesive tissues; black arrows indicate CK-18 positive mesothelial cells or injured mesothelial cell lines). [file 1740317.f1.doc]

**Supplementary** **Tables**

**Supplementary Table 1. Adhesion scoring scheme of** Lauder et al.

| Score | Description |
| --- | --- |
| 0 | No adhesions |
| 1 | Thin filmy adhesions |
| 2 | More than one thin adhesion |
| 3 | Thick adhesion with a focal point |
| 4 | Thick adhesion with planar attachment |
| 5 | Very thick vascularized adhesions or more than one planar adhesion |

**Supplementary Table 2. Adhesion scoring scheme of** Hoffmann et al.

| Grades | Description |
| --- | --- |
| Area scoring scheme | |
| 0 | No adhesion |
| 1 | Cecum to bowel adhesion |
| 2 | Cecum to sidewall adhesion in less than 25% of the abraded surface area |
| 3 | Cecum to sidewall adhesion spanning between 25 and 50% of the abraded surface area |
| 4 | Cecum to sidewall adhesion spanning over 50% of the abraded surface area |
| Area scoring scheme | |
| 0 | No adhesion |
| 1 | Gentle traction required to break adhesion |
| 2 | Blunt dissection required to break adhesion |
| 3 | Sharp dissection required to break adhesion |
| Area scoring scheme | |
| 0 | No adhesion |
| 1 | Filmy adhesion |
| 2 | Vascularized adhesion |
| 3 | Opaque or cohesive adhesion |

**Supplementary Table 3. Histopathological criteria for adhesion scoring**

| Score | Degree of fibrosis | Degree of inflammation |
| --- | --- | --- |
| 0 | None | No inflammation |
| 1 | Mild | Giant cells, lymphocytes, and plasma cells |
| 2 | Moderate | Giant cells, plasma cells, eosinophils, and neutrophils |
| 3 | Severe | Inflammatory cell infiltration and  microabscess formation |


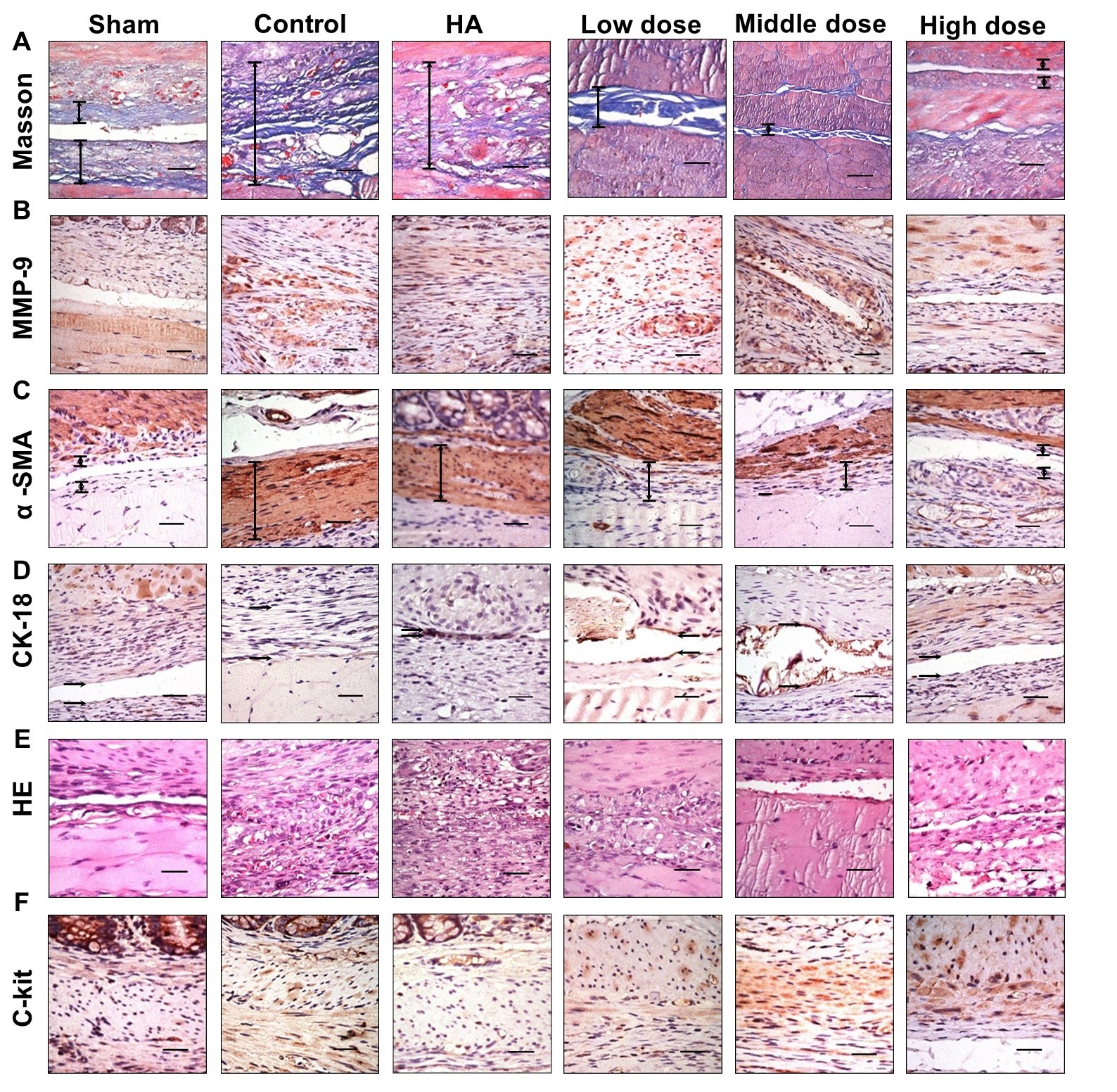


**Supplementary Figure 1. Immunohistochemical staining of Masson, MMP-9, α-SMA, CK-18, HE and C-kit of the different groups.** The magnification is 200× (double-headed black arrows indicate adhesive tissues; black arrows indicate CK-18 positive mesothelial cells or injured mesothelial cell lines).
